# Multi-Environment QTL Mapping of Rust Resistance in Faba Bean (*Vicia faba* L.) to *Uromyces viciae-fabae*

**DOI:** 10.3390/plants14182860

**Published:** 2025-09-13

**Authors:** Sergio G. Atienza, Amero A. Emeran, Ramadan A. Arafa, Fouad Maalouf, Josefina C. Sillero, Carmen M. Ávila

**Affiliations:** 1 Instituto de Agricultura Sostenible-CSIC, Avenidal Menéndez Pidal s/n, 14004 Córdoba, Spain; 2 Faculty of Agriculture, Kafrelsheikh University, Kafr El-Sheikh 33516, Egypt; emeranaa@gmail.com; 3 Plant Pathology Research Institute, Agricultural Research Center, Giza 12619, Egypt; arafa.r.a.85@gmail.com; 4 Biodiversity and Crop Improvement Program (BCIP), International Center for Agricultural Research in the Dry Areas (ICARDA), Terbol, Lebanon; 5 Área Mejora Vegetal, Instituto Andaluz de Investigación y Formación Agraria, Pesquera, Alimentaria y de la Producción Ecológica-Centro Alameda del Obispo, Avda. Menéndez Pidal s/n, 14004 Córdoba, Spain; josefinac.sillero@juntadeandalucia.es

**Keywords:** DArTSeq, faba bean, Hedin genome, *Uromyces viciae-fabae*, QTL mapping, resistance, RILs, BPL-261, slow rusting, marker-assisted selection

## Abstract

Faba bean rust is one of the major threats to the cultivation of faba beans worldwide. Three genes for rust resistance (*Uvf-1*, *Uvf-2* and *Uvf-3*) and fifteen marker-trait associations have been identified so far. This study examines the genetic basis of rust resistance derived from BPL-261, an accessions that exhibits low infection frequency and a long latency period. We constructed a genetic map based on a RIL6 population derived from the BPL-261/Vf-274 cross, which consists of 91 individuals. Subsequent generations were used to evaluate rust resistance in Lattakia (Syria), Kafr El-Sheikh (Egypt) and Córdoba (Spain). A total of six QTLs for resistance were detected on chromosomes II, III, IV and V. Comparative analyses suggest that the QTL located on chromosome V is likely to be associated with *Uvf-3*. The QTL on chromosome III is close to *Uvf-2* but it seems to be a different QTL since the confidence intervals do not overlap. Finally, the other QTLs constitute additional sources of rust resistance in faba beans. Functional analysis of the candidate genes within the confidence intervals suggests the hypothetical involvement of various resistance mechanisms, with salicylic acid playing a significant role but it should be confirmed with further studies. Our results advance understanding of rust resistance in faba bean. Markers identified in this study should be used to develop kompetitive allele-specific PCR (KASP) assays, after their utility has been confirmed in different genetic backgrounds. This will contribute to the development of durable rust-resistant faba bean cultivars.

## 1. Introduction

Faba bean (*Vicia faba* L.) is an important cool-season food legume with worldwide importance. It is one of the oldest cultivated grain legumes [1]. This species is cultivated for its protein content for human and animal consumption, and can be used as a dry bean, green bean or green manure. It also provides ecological benefits for more sustainable agriculture. Firstly, its symbiosis with rhizobia improves soil fertility by fixing atmospheric nitrogen, reducing the need for fertilizers and mitigating the environmental impact of N_2_O emissions. Secondly, it plays an important role in mitigating pests and diseases, being regarded as one of the most effective options for breaking the cycle of pests and diseases in rotation with wheat [2].

The leading producers of faba bean are China, Ethiopia, the United Kingdom and Australia. These countries account for 57% of global faba bean production, including both dry and green beans [3]. Faba beans has declined in Europe over the last few decades [4].

The cultivation of faba bean is affected by abiotic stresses, such as high temperatures or drought, as well as biotic factors, including pests such as aphids and parasitic weeds (e.g., broomrape), and diseases such as rust, ascochyta blight and chocolate spot. One of the major threats to faba bean is the incidence of rust, caused by the fungus *Uromyces viciae-fabae* (Pers.) J. Schröt. Faba bean rust is the most common disease in New South Wales and Queensland, which are the main sites for faba bean cultivation in Australia [5]. It is also a major disease in Europe, China, North Africa and the Middle East, where it can produce moderate to substantial yield losses [6,7]. However, when infection occurs early in the season, yield losses of up to 70% can occur (reviewed by [8]. *Uromyces viciae-fabae* is a macrocyclic autoecious rust that produces all five spore forms on a single host (reviewed by [9]. Although there is limited information about this pathogen, sexual recombination is frequent, which increases the likelihood of variation. Indeed, the existence of different *U. viciae-fabae* races has been suggested [10].

Control methods for faba bean rust include biological and chemical control, as well as disease resistance [11]. However, disease resistance is the most effective method to control diseases including rust and can reduce the impact of pesticides. Two types of resistance to rust have been described in faba bean: one with incomplete non-hypersensitive resistance and the other with incomplete resistance and late hypersensitivity associated with late-acting necrosis of the host tissue [12]. Both result in reduced disease severity and area under the disease progress curve (AUDPC) [12]. A collection of 648 faba bean accessions allowed the identification of new sources of resistance, including the accession BPL-261, which exhibits non-hypersensitive, slow-rusting resistance [12]. Other accessions characterized in this study were 2N-52 and V-300. Both accessions exhibited incomplete resistance with late hypersensitivity and have been utilized in prior research to determine the genetic basis of rust resistance. Indeed, accession 2N-52 was used as the resistance donor to identify the major *Uvf-1* gene [13]. Another couple of single dominant genes derived from a single plant selection from cultivar ‘Doza’ and Ac1655 (V-300), were identified [14]. Doza#12034 also exhibits necrosis upon inoculation with pathotype 24–40 [5]. Developing RIL populations from the crosses between Fiord and Doza#12034 (F_6_) and Fiord and Ac1655 (V-300) (F_4_) has allowed the identification of the *Uvf-2* and *Uvf-3* major genes, respectively, on chromosomes III and VV under controlled conditions against a single pathotype [5].

While both types of rust resistance described in faba bean by Sillero et al. [12] resulted in reduced disease severity and AUDPC, only the accession BPL-261 exhibited low disease severity and AUDPC values due to low infection frequency and a long latency period, among the 648 accessions tested [12]. However, the genetics of non-hypersensitivity, slow-rusting resistance have not yet been studied, despite this offering the potential to provide new tools for developing durable, rust-resistant faba bean cultivars. Identifying new QTLs for rust resistance would create new opportunities for pyramiding resistance genes into elite cultivars.

The aim of this study is to identify the genetic basis of rust resistance, which would be of interest to faba bean breeders.

## 2. Results

### 2.1. Construction of the Genetic Map

A RIL population (F_6_) consisting of ninety-one individuals was developed from the BPL-261/Vf-274 cross and used for map construction. Subsequent generations were then evaluated for rust resistance in field trials in Lattakia (Syria) in 2010, Kafr El-Sheikh (Egypt) in 2016 and Córdoba (Spain) in 2016, 2021 and 2022 to account for potential pathogen diversity. The mapping population was genotyped using the DArTSeq platform (Diversity Array Technology Pty Ltd., DArT P/L, Canberra, Australia). An initial dataset of 10,775 markers was considered for map construction, after filtering out those markers with a call rate below 90% and missing data exceeding 7.5%. Markers deviating from Mendelian segregation were also excluded during map construction. The initial set of markers included 7578 silico DArT, 3292 SNPs. The final map contained 628 unique positions (bin markers) distributed in six linkage groups (Table 1).

The map spans 1320 cM which makes for an average inter-marker distance of 2.1 cM. The inter-marker distance ranged between 0.9 for chromosome IV and 4.9 cM for chromosome I, where the largest gap in the map (19.1 cM) was found (Table 1).

### 2.2. Alignment to Hedin Genome

The DArTSeq marker sequences were aligned to the Hedin genome using the IPK Galaxy Blast Suite (https://galaxy-web.ipk-gatersleben.de/, accessed on 10 July 2025). A second alignment was performed using the DArTSeq sequences as queries against the coding loci and complete CDs from the Hedin2 annotation data (downloaded from https://projects.au.dk/fabagenome/genomics-data, accessed on 16 October 2024), employing a Blastn search [15] as coded in BLAST+ [16] (Supplementary File S1). These results enabled the linkage groups to be assigned unequivocally to specific faba bean chromosomes.

**Figure 1:**
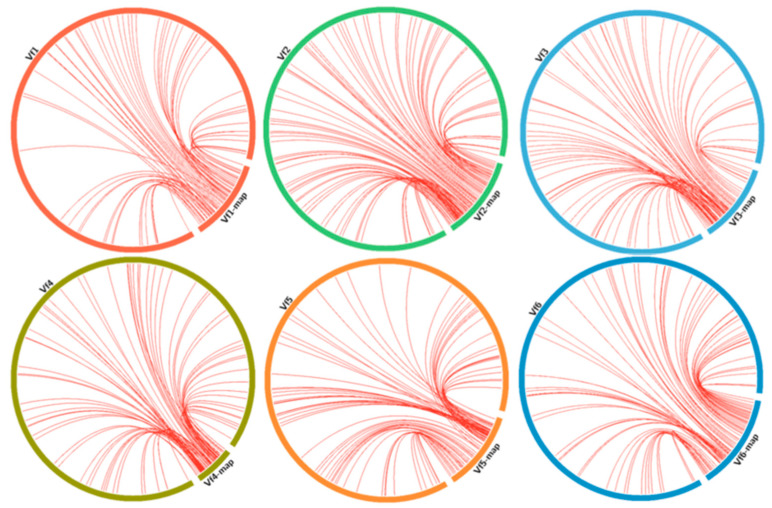
Collinearity between BPL-261/Vf-274 genetic map and ‘Hedin’ assembly.

The collinearity between the genetic map and the physical position in the Hedin assembly was investigated using the alignment results (Figure 1). Spearman’s rank correlations were calculated between the relative order of markers in the BPL-261/Vf-274 map and the relative position of the faba bean sequences in the Hedin pseudomolecules obtained after the BLASTn analysis.

Good collinearity was observed between the genetic map and the physical positions, with Spearman-rank correlations above 0.7 in all cases except for chromosome III (0.68).

### 2.3. QTL Detection

**Figure 2:**
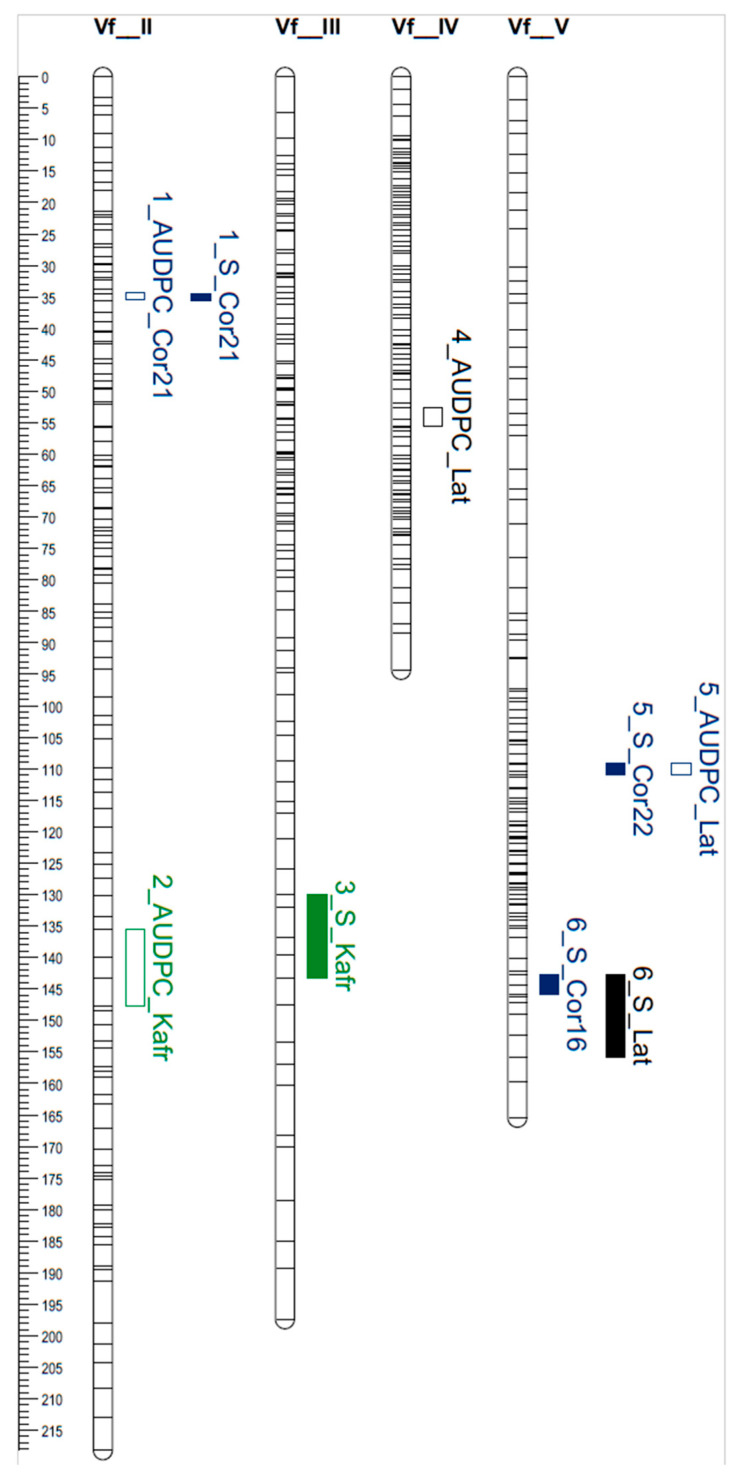
Genetic map of the BPL-261/Vf-274 population (genetic distance in cM is shown at left) and QTL mapping for rust resistance in faba bean. AUDPC: Area Under Disease Progression Curve; S: Severity; Cor: Córdoba; Lat: Lattakia; Kafr: KafrEl-Sheikh.

The RIL population was evaluated for rust resistance in three different Mediterranean locations: Lattakia (Syria, 2010), Kafr El-Sheikh (Egypt, 2016) and Córdoba (Spain, 2016, 2021 and 2022). The severity of rust resistance was evaluated in all field trials. We also scored the Area Under the Disease Progress Curve (AUDPC) in Lattakia, Kafr El-Sheikh and Córdoba in 2021. A total of six QTLs for resistance were detected on chromosomes II, III, IV and V and numbered from 1 to 6 (Table 2, Figure 2).

Seven markers within the confidence interval of the QTLs provided significant hits to faba bean genes. The functional annotation of these genes was investigated in EnsemblPlants, but two of them lacked annotation in the PANTHER (Protein Analysis Through Evolutionary Relationships) database (Table 3).

The annotation of these genes corresponds to a homeodomain-leucin zipper class III (HD-ZIP class protein) (chromosome II), a WRKY protein, and an auxin efflux carrier (chromosome IV). Markers for QTL on chromosome V were annotated as YTH protein and Monensin Sensitivity1/Calcium Caffeine Zinc Sensitivity 1 (MON1) and YTH protein (Table 3). All these genes play specific roles in defense/resistance mechanisms in rusts pathosystems as discussed below.

## 3. Discussion

### 3.1. Map Construction and Alignment to Hedin Genome

The map results are comparable to those of the high-density map constructed using the ‘Vfaba_v2′ 60k Axiom array [17], indicating that DArTSeq sequences allow the development of high-density maps in faba bean, as has been achieved for other species in our group [18,19]. In fact, the maximum gap reported for each chromosome in this study is smaller than that reported using the ‘Vfaba_v2′ 60K Axiom array [17]. The 19.1 cM gap indicates a lack of recombination in this region of the mapping population, which is likely due to its limited size. In other studies, large gaps have been associated with the location of markers exhibiting distorted segregation [17]. However, this is not the case here, as distorted markers were excluded prior to linkage analysis in the current study. Nevertheless, further studies with larger population sizes would help to refine the position of the QTLs and their effects.

The collinearity results are comparable to those recently reported [17], although we did not found the large gaps reported for chromosomes IV and V in that study. Chromosome III showed the lowest level of collinearity. This could be due to structural rearrangements in this chromosome, or to assembly issues in the Hedin genome.

### 3.2. Functional Analyses

Only the QTLs on chromosome V were effective in different areas (Córdoba and Lattakia), while the other QTLs were exclusive to one location. This may indicate the existence of different pathogen races, as previously suggested [10]. The existence of different races is an important issue in other crops, such as wheat. Initiatives such as the RustWatch (https://agro.au.dk/forskning/projekter/rustwatch/, accessed on 15 July 2025) have been established to monitor the diversity of rust pathogens in wheat across seasons. It seems likely that *U. viciae-fabae* is a complex pathogen comprising several co-existing races, but further research is needed to confirm this. Investigating the pathogen in more depth and monitoring it across seasons and regions would deepen our understanding of the genetic basis of rust resistance in faba bean. Further research is needed to develop higher-yielding cultivars that are more adaptable to sustainable agriculture.

The giant genome of the faba bean (around 13 Gb) has posed a significant challenge in generating high-quality genome assemblies. This has limited our ability to investigate the molecular basis of traits of interest. However, the publication of the first high-quality, chromosome-scale faba bean genome [20] is a major step towards exploiting this species’ potential.

The WRKY transcription factors play an important role in several signaling cascades and regulatory networks, with implications for plant defense [21]. Recently, TaWRKY27 was shown to be a pathogen-associated susceptibility gene involved in wheat-*Puccinia striiformis* interactions, promoting susceptibility. Silencing this gene increases resistance, whereas overexpressing it promotes rust susceptibility in wheat [22]. Overexpression of this gene also promotes auxin accumulation, which suppresses the expression of defense-related genes [22].

HD-ZIP proteins are a class of transcription factors that are unique to plants [23]. HD-ZIP class III transcription factors are associated with auxin distribution and play a key role in vascular tissue differentiation due to changes in the auxin signaling pathway (reviewed by [23]. The third gene codes for an auxin efflux carrier which is important for directing auxin fluxes [24]. Therefore, these three genes appear to be involved in the signaling and regulation of auxin. This is relevant, as auxin has been shown to interfere with SA-mediated defense and promote physiological changes that favor the pathogen proliferation in plants (reviewed by [25].

Salicylic acid (SA) plays an important role in faba bean rust resistance. Indeed, exogenous applications of salicylic acid and benzothiadiazole (a salicylic acid analog) induces systemic acquired resistance in faba bean, producing a lower degree of infection [26]. This demonstrates the importance of SA in faba bean resistance to rust. Salicylic acid suppresses auxin signaling by down-regulating auxin-responsive genes or up-regulating GH3 enzymes, which are responsible for regulating auxin homeostasis (reviewed by [25]. Conversely, auxin signaling represses SA levels and signaling [27], which is detrimental to SA-mediated defense. Furthermore, auxin can promote physiological changes that favor the pathogen growth in the plant, such as the positive regulation of expansins, which are involved in cell wall loosening [25].

The other two candidate genes associated with the QTLs identified in this work on chromosome V correspond to a Monensin Sensitivity1/Calcium Caffeine Zinc Sensitivity 1 (MON1) and a YTH protein (Table 3), which are related to plant’s defensive response.

Firstly, MON1 plays a significant role in defending against the biotrophic fungus *Blumeria graminis* in barley [28]. Specifically, the effector CSEP0162 targets MON1 to inhibit encasement around haustoria and biotrophic hyphae, constricting pathogen growth [28]. MON1 is also required for Arabidopsis resistance to powdery mildew [28]. These findings demonstrate the conservation of the MON1-related resistance mechanism across species, supporting its potential role in the faba bean/rust pathosystem.

The last candidate gene is annotated as YTH protein. YTH proteins contain methyl-binding aromatic pockets that recognize and bind *N^6^*-methyladenosine (m^6^A) marks [29]. Post-transcriptional regulation of mRNA is crucial for gene regulation and maintaining genome stability, and m^6^A is the most prevalent internal covalent mRNA modification known in eukaryotic transcriptomes [30]. In Arabidopsis, the YTH proteins ECT9 can form condensates with ECT1 to control immune responses [31]. The same authors also demonstrated that the *ect1/9* double mutant dramatically decreases *ECT9* and *ECT1* expression levels, and that ECT9-ECT1 condensates decrease following SA treatment. The involvement of SA in the regulation of YTH proteins suggests a potential role in the faba bean/rust pathosystem, as has been described in other species. Indeed, the YTH protein family has been analyzed at genomic and transcriptomic levels in wheat [32]. Transcriptomic analysis revealed that TaDF proteins, which share the YTH domain, were upregulated upon infection with stripe rust and other pathogens. Further studies analyzing the specific role of YTH proteins in relation to faba bean resistance to rust are needed, focusing on post-transcriptional regulation and the potential involvement of SA.

### 3.3. Comparative Analysis

The potential co-localization of the QTLs identified in this study with previous studies was investigated, taking into account the alignment results (Supplementary File S1) and the KASP markers flanking the *Uvf-2* and *Uvf-3* genes [5]. These KASP marker sequences were used as queries in BLASTn searches at EnsemblPlants to determine their physical position in the faba bean genome (Table 4). The best hits for the *Uvf-2* flanking sequences were found on chromosome III between 1203.37 and 1319.54 Mbp. In the present study, two markers within the confidence interval of the QTL located on chromosome III were aligned to chromosome III at 1468.62 and 1477.52 Mb (Supplementary File S1). Therefore, although it cannot be completely ruled out that the QTL located on chromosome III in this study targets the same region as *Uvf-2*, it seems that they are different since the confidence intervals do not overlap.

The flanking markers for *Uvf-3* were located on chromosome V, between 466.68 and 816.40 Mbp. The candidate genes Vfaba.Heding2.R1.5g088520.1 and Vfaba.Heding2.R1.5g114960 were located at 618.99 and 796.25 Mbp, respectively. Both candidate genes would be within the confidence interval for *Uvf-3*. Therefore, the QTL detected on chromosome V in the present study are likely targeting additional *Uvf-3* gene variation.

Similarly, a seven-parent MAGIC population was used to identify marker-trait associations linked to several agronomic traits, including rust resistance [33]. The authors identified 15 marker-trait associations for rust, five of which were stable across locations. None of these markers overlapped with *Uvf-2, Uvf-3* or any of the QTLs identified in the present work. Nevertheless, the AX-416763140 marker, which was stable across environments, was located on chromosome II at 890.01 Mbp, which is relatively close to the QTL identified on chromosome II at Córdoba. In the present study, marker 3527287 was found to be located less than 4 cM from the QTL on chromosome II, at 904.1 Mbp in the faba bean genome. Therefore, the marker AX-416763140 [33] and the QTL on chromosome II detected in Córdoba in the present study may target the same resistance.

## 4. Materials and Methods

### 4.1. Plant Material and Field Trials

The faba bean accession BPL-261 shows incomplete non-hypersensitive resistance and a long latency period [12], while the accession Vf-274 was selected as susceptible. A RIL6 population was developed by crossing the resistant parent, BPL-261, with the susceptible parent, Vf-274, at the IFAPA-Centro Alameda del Obispo (Córdoba, Spain). The recombinant inbred line (RIL) population derived from the BPL-261/Vf-274 cross was developed by single-seed descent from the F_2_ population. Each generation was grown under controlled conditions to avoid cross-pollination.

Field experiments were conducted under field conditions in three locations: Lattakia (Syria) in 2010, Kafr El-Sheikh (Egypt) in 2016 and Córdoba (Spain) in 2016, 2021 and 2022.

Lattakia: Faba bean genotypes were planted in early January, which is late faba bean sowing in the area, in order to coincide with favorable weather conditions for rust development under natural inoculum. Sprinkler irrigation was used to promote disease development.

Kafr-El-Sheikh: The field experiment was conducted during the 2016 growing season at the experimental farm of the Faculty of Agriculture, Kafr-El-Sheikh University, Egypt (31.0° N, 30.9° E). Seeds were hand-sown in November 2015 on a loamy calcaric Fluvisol (pH 7.5; EC 1.9 dS m^−1^) soil and harvested in late May. The crop was cultivated under organic conditions, without the use of fertilizers or pesticides. Hand weeding was carried out as required. Surface irrigation was applied four times, each delivering 450 m^3^ ha^−1^ of water (pH 7.4; EC 0.39 dS m^−1^): immediately after sowing, in December, in March, and in April. No artificial inoculation was carried out, as faba bean rust occurs regularly under natural field conditions at the site.

Cordoba: Rust resistance was assessed in 2021 and 2022 using a randomized block design with three replications. Ten seeds of each RIL genotype were sown along a 1 m-long row with row spacing of 70 cm. In 2016, each RIL was represented solely by one plant, the one used for DNA isolation.

Average daily temperatures (°C) and monthly precipitation (mm) at each location are shown in Supplementary Figure S1.

### 4.2. Genetic Map Construction

Genomic DNA was isolated from young leaves following the CTAB protocol with slight modifications [34] using TissueLyser II mill (Qiagen), two stainless-steel balls (5 mm diameter) for sample disruption and 2 mL Eppendorf tubes. Ninety-one individuals from the BPL-261/Vf-274 RIL (F_6_) population were used for map construction. Genotyping by sequencing analysis of the mapping population was performed by means of DArTSeq platform at Diversity Arrays Technology Pty Ltd. (Canberra, Australia).

The genetic map was constructed using Joinmap ^®^ v. 5.0 (Kyazma ^®^, Wageningen, The Netherlands) using ninety-one F_6_ individuals. Only DArTSeq markers with a call rate of above 90% and with consistent segregation in the parental lines of the population were used for mapping. Markers deviating from the 1:1 ratio expected for mendelian segregation in a RIL population were excluded. A minimum LOD of 7.0 was used to assign markers to chromosomes. For each chromosome, several rounds of mapping were performed by excluding markers co-segregating in the same positions and using the EML algorithm (fastest). A final round of mapping was performed using the Regression Mapping algorithm (only second round of mapping) and the Kosambi mapping function to obtain the final maps.

Collinearity between genetic map and physical positions in the faba bean genome were inspected using CIRCOS 0.67 [35] based on DArTSeq markers with a significant match to ‘Hedin’ chromosomes after BLASTn alignment (%identity cutoff = 98%, minimum query coverage = 85%) at https://galaxy-web.ipk-gatersleben.de/, accessed on 10 July 2025. In addition to this, a second alignment was performed using the DArTSeq sequences as queries against the coding loci and complete CDs from Hedin2 annotation data https://projects.au.dk/fabagenome/genomics-data (accessed on 10 July 2025) using a Blastn search [15] and BLAST+ 2.6.0 [16] with the following criteria: Evalue of <2.5 × 10^−7^ and sequence identity of > 80%.

### 4.3. Resistance Scoring

The RIL population was evaluated in different years and locations. Specifically, the ninety-one RIL families used to develop the genetic map presented in this study were evaluated under natural infection in field conditions in three locations: Lattakia (Syria) in 2010, Kafr El-Sheikh (Egypt) in 2016 and Córdoba (Spain) in 2016, 2021 and 2022. A completely randomized design was employed. The number of replications depended on the available seed number at any given time. Thus, two replications were conducted in Syria and Egypt and three in Córdoba 2021 and 2022. The exception was Córdoba in 2016, where phenotypic data corresponded to plants collected for DNA extraction, genotyping, and map construction. In all other environments, each family in each replication was represented by a single 1 m row with a maximum of ten plants spaced 10 cm apart. Entries were separated by 0.7 m and a susceptible check was introduced into the design as spreader. In the trials in Egypt and Spain, the percentage of foliar area covered by the disease was scored, whereas a 0–9 scale was used in Syria as follow: 1 = no pustules or very resistant small non-sporulating flecks; 3 = few scattered pustules covering less than 1% of leaf area, few or no pustules on stem; 5 = pustules common on leaves, covering 10–40% of leaf area, some pustules on stem; 7 = pustules very common on leaves, covering 40–80% of leaf area, many pustules on stem; 9 = extensive pustules on leaves, petioles, and stem covering 80–100% of leaf area, many leaves dead and plant defoliated. Where conditions were adequate, scoring was performed several times throughout the disease development which enabling us to calculate the Area Under the Disease Progress Curve (AUDPC) in Syria 2010, Egypt 2016 and Spain 2021.

### 4.4. QTL Analysis

Average disease scores for each trial were used for QTL analysis with for QTL analyses with MapQTL ^®^ v. 6.0 (Kyazma ^®^, Wageningen, The Netherlands). The nonparametric Kruskal–Wallis test was used to identify marker-trait association in a first stage. After this, interval-mapping analyses were carried out [36,37]. Finally, rMQM mapping was performed [38,39,40]. The QTL significance (*p*-value) was calculated by using a permutation analysis (1000 permutations) [41]. QTL figures were generated by using MapChart software v2.32 [42]. The use of *p* values was reported as continuous quantities following the recommendations in [43]. Uncertainty in the QTL position was estimated using a 2-LOD support interval [37]. The QTLs were named using the trait abbreviation followed by the location and year.

## 5. Conclusions

Comparative analyses suggest that the QTL located on chromosome V is likely to be related to *Uvf-3*. The novel QTLs on chromosomes III, IV and V constitute new sources for rust resistance in faba bean. Functional analysis of the candidate genes suggests the hypothetical involvement of various resistance mechanisms, with salicylic acid playing a significant role, but it should be confirmed with further studies. The current results advance the understanding of rust resistance in faba bean. Further steps should include the development of kompetitive allele-specific PCR (KASP) assays for marker-assisted selection, after their utility has been confirmed in different genetic backgrounds. These findings will contribute to the development of durable, rust-resistant faba bean cultivars.

## Figures and Tables

**Table 1:** Summary of the BPL-261/Vf-274 genetic map.

Chrom.	Number of Bin Markers	Total Distance (cM)	Average Distance (cM/Marker)	Maximum Gap (cM)
I	86	418.4	4.9	19.1
II	125	218.2	1.7	6.6
III	108	197.4	1.8	8.5
IV	104	94.2	0.9	5.8
V	101	165.5	1.6	6.1
VI	104	226.8	2.2	6.5

**Table 2:** QTL for rust resistance in the BPL-261/Vf-274 RIL population.

QTL	QTL_Name ^1^	Chm	LOD	Position (cM)	Flanking Markers	*p*-Value ^2^	R ^2^ (%)
1	AUDPC_Cor21	II	5.47	34.6	13909843/13863183	0.001	28.5
1	S_Cor21	II	6.63	34.4	13909843/13863183	0.001	31.0
2	AUDPC_Kafr	II	3.20	143.2	3527667/13913387	0.048	14.5
3	S_Kafr	III	3.37	137.9	13868837/13891645	0.0382	15.2
4	AUDPC_Lat	IV	4.59	54.4	13904768/13913479	0.0022	20.2
5	S_Cor22	V	4.86	109.4	3518937/13908777	0.0024	24.3
5	AUDPC_Lat	V	3.65	110.5	3518937/13908777	0.0355	15.4
6	S_Cor16	V	2.81	144.1	3526504/3524870	0.1563	14.2
6	S_Lat	V	3.22	143.8	3526504/13911090	0.0746	37.8

^1^ AUDPC: Area Under the Disease Progress Curve; S: Severity. Cor: Córdoba; Kafr: Kafr El-Sheikh; Lat: Lattakia. ^2^ QTL probability determined from the permutation test.

**Table 3:** Functional analysis.

Marker	Position (cM)	Chrom	Gene	Annotation at PANTHER ^1^
3521015	143.2	II	Vfaba.Hedin2.R1.2g194840	Class III homeodomain-leucine zipper family
13913387	147.8	II	Vfaba.Hedin2.R1.2g210080	PTHR47932
3520124	54.4	IV	Vfaba.Hedin2.R1.4g162080	WRKY
13913479	55.6	IV	Vfaba.Hedin2.R1.4g212280.1	Auxin Efflux Carrier
13908777	111.0	V	Vfaba.Hedin2.R1.5g088520.1	YTH domain containing protein
3526504	142.8	V	Vfaba.Hedin2.R1.5g187960	PTHR23346
3516136	149.0	V	Vfaba.Hedin2.R1.5g114960	Regulator of MON1-CCZ1 complex

^1^ PANTHER (Protein Analysis Through Evolutionary Relationships) annotation.

**Table 4:** BLASTn results for KASP markers flanking *Uvf-2* and *Uvf-3*.

Gene	Flanking Marker ^1^	Distance (cM)	Gene (Best Hit) ^2^	Best Hit Position (Mb) ^2^
*Uvf-2*	KASP_C250539	2.5	None	1203.37
	KASP_Vf_0703	10.1	Vfaba.Hedin2.R1.3g174360.1	1319.54
*Uvf-3*	KASP_ACxF165	2.9	Vfaba.Hedin2.R1.5g117640.1	816.40
	KASP_Vf_1090	4.9	Vfaba.Hedin2.R1.5g073360.1	468.97
	KASP_Vf_1090	4.9	Vfaba.Hedin2.R1.5g072920.1	466.68

^1^ KASP markers flanking *Uvf-2* and *Uvf-3* [5]. ^2^ BLASTn results at EnsemblPlants.

## Data Availability

Data are contained within the article and Supplementary Materials.

## Supplementary Materials

The following supporting information can be downloaded at https://www.mdpi.com/article/10.3390/plants14182860/s1: Supplementary File S1. Contain Tables S1–S6: Atienza et al. Genetic map (chrm I-VI) for the BPL-261/Vf-274 RIL population and alignment results to faba bean genome. Figure S1. Average temperatures and precipitations at each location and year.

## Abbreviations

The following abbreviations are used in this manuscript:

AUDPC	Are Under the Disease Progress Curve
MON1	Monensin Sensitivity1/Calcium Caffeine Zinc Sensitivity 1
PANTHER	Protein Analysis Through Evolutionary Relationships
RIL	Reconbinant Inbred Lines
SA	Salicylic acid
